# The NISE Net: Bringing the Study of the Very Small to US Science Centers

**DOI:** 10.1371/journal.pbio.0050283

**Published:** 2007-10-16

**Authors:** Margaret Glass

## Abstract

With funding from NSF, the Nanoscale Informal Science Education Network aims to use informal learning experiences to engage families, schoolchildren, and adults in nanoscale science, engineering, and technology in science centers and museums across the US.

Nanoscale science has been in the research labs of industry and academia for over 20 years. Consumers increasingly confront an abundance of products that contain nanoparticles or that claim to be a result of nanotechnology, including skin-care products, wrinkle- and stain-resistant fabrics, and sports items like golf clubs and baseball bats [1]. Yet as recently as late 2006, over 80% of the American public reported that they had heard nothing or next to nothing about nanotechnology [2].

The Nanoscale Informal Science Education Network (NISE Net) is an ambitious initiative funded by the National Science Foundation (NSF) to engage families, schoolchildren, and adults in nanoscale science, engineering, and technology through informal learning experiences in science centers and museums across the United States. NISE Net has designed exhibits and public programs about nanoscience and plans to disseminate these to 100 science centers and museums by the year 2010. Two years into this multi-year project, the team has reached an inflection point—a time to assess critically the status of our work, re-evaluate our original assumptions, and redouble our efforts to support the collective vision that has emerged from this period of intensive collaboration.

## Nanoscale Science

The prefix “nano” derives from the Greek “nanos,” meaning “dwarf.” A nanometer is one-billionth of a meter (10^−9^ m); a human hair is about 100,000 nanometers thick. Colloquially, nano is sometimes used as shorthand for nanoscale science and technology, or the science of the very small (though its recognition as a brand-name digital audio player is far more widespread).

Nano research and technology crosses many classical disciplinary boundaries between biology, chemistry, physics, material science, and engineering. Unifying these diverse disciplines is the focus on the structure and dynamics of matter at the molecular level—the level at which things are built in nature. At the nanoscale, matter exhibits different physical characteristics than it does at the macroscale. The relative importance of basic physical forces also changes. For example, the increased surface area of particles at the nanoscale accelerates chemical reactions; gravity becomes less important, and electrostatics, friction, and heat from kinetic energy assume greater relevance.

The attributes of smallness can lead to unexpected new observations in familiar materials. For example, gold and silver exhibit colors differently as nanoparticles than they do in bulk, glowing red or yellow because of the nanoparticles' light-scattering properties. Materials at the nanoscale may be stronger, more water-soluble, or conduct electricity better than the same material at the macroscale.

Manipulating matter at the nanoscale provides new opportunities and challenges in manufacture and engineering. One result of this kind of manipulation is “geckel,” a material that combines the adhesive qualities of nanoscale structures on gecko feet with a synthetic polymer that simulates the proteins that mussels use to cling to underwater surfaces. This hybrid material is an adhesive with immense strength that is functional in both wet and dry environments and is capable of reuse [3]. This is just one of numerous examples of the engineering creativity that is possible when scientists can work at the scale of nature's building blocks. As important as these properties of the nanoscale are for developing novel applications, they leave us with an educational misalignment: whereas Newtonian laws remain the familiar educational entrée into physics, the less familiar laws of quantum mechanics governing the nanoworld now rule leading-edge science.

## The Challenge of Teaching Nano

Research on nanoscale science and technology was established as a federal initiative (National Nanotechnology Initiative, or NNI) in 2001. A 2004 strategic plan set the initiative's current directions. In addition to funding research, the NNI supports a substantial education program on nanoscience and technology for Kindergarten though 12th grade students, university and vocational students, and the public. Since the introduction of the No Child Left Behind act, formal education systems have been under tremendous pressure to create specific curriculum standards and performance objectives that are aligned with structured assessments. This emphasis on structure comes at the expense of flexibility; it has become more difficult to introduce new subject materials into the curriculum. Without special effort, nanoscale science and engineering are not likely to move quickly into the mainstream of formal education. In 2004, NSF funded a national center for learning and teaching nanoscale science and engineering education to begin to address the formal education needs.

In funding NISE Net in 2005, NSF added the important dimension of public engagement and education to the nanoscale science and engineering education effort. Simultaneously, it created a unique opportunity for the informal science education (ISE) community. Science centers and museums represent a significant sector of the ISE infrastructure and attract a large and diverse public. The Association of Science-Technology Centers (ASTC) estimated in its 2006 survey that over 58 million people visited ASTC's 344 US member institutions. Thirty percent of Americans say they have visited a science center or museum in the past 12 months [4].

The NISE Net team made the case for funding science museums to do this work in their original proposal to NSF. “Informal science institutions are adept at making science accessible, approachable, and engrossing. They are experienced at revealing science and scientific inquiry as fundamental human endeavors, with direct impacts on culture and society. They have pioneered new approaches to reaching communities historically underrepresented in the sciences. They have developed powerful methods for engaging adults in discourse and deliberation around the societal implications of science. They have developed innovative media-based methodologies that allow the public to explore science and technology following their own paths of inquiry.” In its effort to bring nanoscience to the public, NISE Net tapped these ISE qualities, which reward the public's trust in science centers and museums as nonpartisan, objective sources of information.

Lead institutions for NISE Net include the Museum of Science, Boston (Massachusetts), the Exploratorium (San Francisco, California), and the Science Museum of Minnesota (Saint Paul, Minnesota). From the beginning, this core team was joined by an array of ten organizational partners, 23 “thinking” partners, and 12 advisors drawn from informal science and nanoscience research organizations (NISE participants). This nascent network intends to spread its reach well beyond the original cohort. In addition to developing nanoscience-based exhibits and program, NISE Net aims to produce a sustainable network of relationships and alliances across the ISE community and between the ISE community and the nanoscience research community. This link with nanoscientists is especially important. One observation that has consistently emerged from evaluations of science communication programs is the importance of providing the public with a direct link to real scientists. With that in mind, NISE Net continues to cultivate relationships with researchers and seeks to incorporate them into activities and exhibits in visible and accessible ways.

## NISE Net Activities and Products

Exhibits and programs are the signature products of many ISE projects. Front-end research and evaluation of previous nano exhibits helped to inform NISE Net decisions about the nature of content to convey and the kinds of components to test [5]. By the middle of the second year, the NISE Exhibits and Programs Team had developed five thematic exhibit packages: Introduction to Nano; Nanomedicine; Nano in Energy and the Environment; a Nano Lab; and Nano Images. Prototypes of these exhibit packages were evaluated in March, 2007, by over 580 visitors at the Science Museum of Minnesota. A workshop for NISE Net partners was held in that same month, allowing for review of the prototypes by a group of over 100 co-workers and peers, and leading to further revisions and refinements.

The team also prototyped about 20 programs. Designed to be presented as short shows or demonstrations, these programs range from relatively didactic presentations with a firm footing in the research realm, to the classic, hands-on shows given at science centers (concrete representations of complex concepts, using familiar everyday materials and incorporating audience volunteers). More innovative programs include game show recreations with nano content and skits commissioned from acting groups. As a result of critical evaluation, these initial efforts have been winnowed down to a smaller subset of programs that are now circulating and being presented in “beta” testing mode. The revised exhibits and programs were made available to the broader ISE professional community at the annual ASTC conference in October, 2007, at the California Science Center in Los Angeles. Beginning in 2008, NISE Net exhibit packages and programs will be disseminated to other science centers.

Another NISE Net team was charged with the creation of public forums intended to engage adults and older youth by incorporating dialogue and deliberation around societal implications of nanoscale science, engineering, and technology. NISE Net forums to date have engaged the public in discussions about what role the public should play in decisions about nanotech policy and about risks associated with consumer skin-care products containing nanoparticles and with new medical (nano) technologies. NISE Net is increasing collaboration with other organizations with complementary goals, such as the Center for Nanotechnology in Society led by Arizona State University, also funded by NSF.

A NISE Net media team has been experimenting with different media formats for public engagement. Examples of current products include a multimedia kiosk on nanomedicine and short films and videos, ranging from features on nanoscientists and their work to a YouTube.com post on alternative ways of describing the size of a nanometer. In a complementary effort, the NISE Visualization Laboratory has been working to identify and develop effective visualization strategies that enhance the public engagement toolkit. This involves expansion of NISE Net partners into the visual arts world to help communicate about the nature and characteristics of the nanoworld.

Professional development is layered throughout NISE Net activities, in keeping with the project goal of enhancing the ISE field's ability to present nano content. The anchor for this capacity-building effort will be a project website (http://www.nisenet.org) that will double as a professional resource center and public portal for NISE Net. The professional development effort also reaches out to the nanoscience research community through the NEO (Nanoscale Education Outreach) program. NEO workshops are offered to graduate students, post-docs, and education directors of nanoscale science, engineering, and technology research centers. The NEO program offers researchers the opportunity to learn how to communicate their science directly to the public by designing and implementing inquiry-based science lessons and activities.

## First Lessons—and Next Steps

In the first two years of this project, it has become apparent that the challenges of conveying information about the nanoworld are real and persistent. It remains stubbornly difficult to convey the absolute size of the nanoworld (perhaps not unexpectedly, this is a problem shared by researchers who work at the other end of the size spectrum—astronomers). It has also become clear that it is hard to avoid text-heavy labels when providing explanations for exhibits about nano. Conveying information about nano is most successful when facilitators interact with and explain underlying phenomena to visitors.

Despite these challenges, NISE Net has found that exhibits and programs appropriate for science centers and museums can indeed be developed to engage the public in nanoscale science and technology. Although visitors may not gain an intuitive understanding of the size of a nanometer, some of the prototype exhibits do help visitors understand that size affects properties and that things at the nanoscale are really, really, small. In the testing during the first two years, visitors more consistently understood the applications of nanoscience to technology than they understood the fundamentals of nanoscale science (e.g., atomic structure and forces), and they were more likely to engage in exhibits if the activities seemed to be fun or relevant to their lives [6]. In addition to familiar exhibit and program techniques, NISE Net has learned about more novel techniques, such as using haptic devices to allow visitors to “feel” nano phenomena and using dialogue and deliberation to engage visitors in exploring issues of relevance.

NISE Net partners have also benefited from managing interactions and collaborations within a large community of practitioners. One byproduct of the rigorous evaluation that has accompanied this work is the realization that the partners involved value the relationships both with each other and with nano researchers as much as they value the specific exhibit, program, or project for which they were contracted. It is precisely the value placed on these relationships that may allow this network to transcend the nanoscale project and provide participants with the means to continue to build capacity and knowledge within their own institutions.

The next few years will be busy ones for NISE Net partners. In the first two years of the project, a team of institutions was pulled together to focus on creating exhibits, programs, media, forums, and other educational products that could effectively engage the public in learning about nanotechnology in science centers and museums. The focus is now shifting from creating those materials to delivering the educational experience in many locations. Already some collective events are being planned that will help collaborating science centers find new ways to bring nanoscience to their visitors across the US.

**Figure 1:**
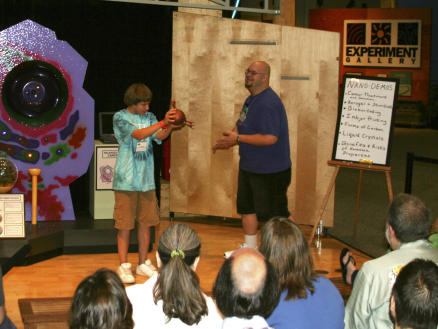
Gallery Demonstration NISE Net program developer Jonathon Mills from the Fort Worth Museum of Science and History presents a program on applications of nanotechnology in cancer detection and treatment. (Image Credit: NISE Network)

**Figure 2:**
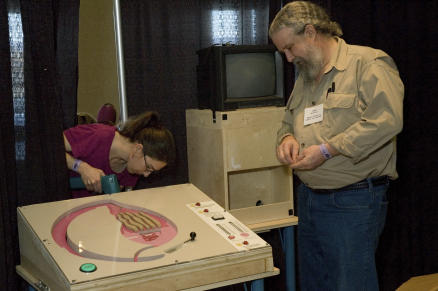
Workshop Setup NISE Net exhibit developers Jenny Gardner and Bob Reynolds from the Oregon Museum of Science and Industry prepare their exhibit prototype for display at a NISE Net Exhibit and Programs Workshop. The prototype explains the use of gold nanoshells in cancer treatment. (Image Credit: NISE Net)

## Abbreviations

ISE: informal science education
NEO: Nanoscale Education Outreach
NISE Net: Nanoscale Informal Science Education Network
